# The efficacy and safety of acupuncture in the treatment of taste disorders after rehabilitation from COVID-19: A protocol for systematic review and meta-analysis

**DOI:** 10.1097/MD.0000000000031649

**Published:** 2022-11-25

**Authors:** Yuqiao Chao, Lili Zhang, Qiaobin Chen, Biwei Chen, Yannan Yu, Shaozong Chen

**Affiliations:** a Shandong University of Traditional Chinese Medicine, Jinan, Shandong, China; b Institute of Acupuncture-moxibustion, Shandong University of Traditional Chinese Medicine, Jinan, Shandong, China.

**Keywords:** COVID-19, meta-analysis, protocol, systematic review, taste disorder

## Abstract

Since the outbreak of the new crown pneumonia epidemic, although it has had a serious impact on people’s lives and health in itself, the sequelae that accompany coronavirus disease 2019 (COVID-19) have also had a serious impact on people’s mental health and quality of life. Taste disorder (TD) is one of the sequelae of COVID-19. Patients may experience reduced or even absent taste sensation, which seriously affects their real life. The efficacy of acupuncture in the treatment of taste disorders has been well reported, but there is a lack of evidence-based medical evidence. Therefore, this study set out to systematically evaluate the efficacy and safety of acupuncture in the treatment of post-COVID-19 taste disorder.

**Methods:**

According to the retrieval strategies, randomized controlled trials on the acupuncture for COVID-19 TD were obtained from Cochrane Central Register of Controlled Trials, Embase, PubMed, Web of Science, the Chinese National Knowledge Infrastructure, the Chinese Biomedical Literature Database, the Chinese Scientific Journal Database and the Wanfang Database, regardless of publication date, or language. Studies were screened based on inclusion and exclusion criteria, and the Cochrane risk bias assessment tool was used to evaluate the quality of the studies. The meta-analysis was performed using Review Manager (RevMan 5.4) and StataSE 15.0 software. Ultimately, the evidentiary grade for the results will be evaluated. This systematic evaluation protocol is registered in PROSPERO under the registration ID CRD42022364653.

**Results:**

The results of this meta-analysis will be submitted to a peer-reviewed journal for publication.

**Conclusion:**

This meta-analysis will evaluate the effect of acupuncture and moxibustion on TD caused by sequelae of COVID-19, providing evidence as to the treatment in these patients.

## 1. Introduction

Novel coronavirus pneumonia has attracted widespread attention since its outbreak and has had a profound impact on both society and the lives of individuals. in April 2020, the Centers for Disease Control and Prevention identified taste disturbance as one of the 6 additional symptoms of neo-coronavirus infection.^[1]^ It was found that neo-coronavirus pneumonia should be highly suspected in patients who develop taste disorders despite having no apparent cause.^[2]^ It was found that most patients with coronavirus disease 2019 (COVID-19) had taste disorders that improved with the control of COVID-19, but some patients still had remission of other clinical symptoms without improvement of taste.^[3]^ Lechien et al^[4]^ conducted a count of 417 patients with mild and moderate neocoronary pneumonia from 4 European countries (France, Belgium, Italy, Spain) and It was found that a large percentage of patients (89%) had taste disorders associated with them, and that some of them continued to show taste disorders after other symptoms had resolved. a study by Ercoli et al^[5]^ concluded that taste disorders are one of the sequelae of patients with COVID-19. In addition, Taste disorders are often present with olfactory disorders, but Lechien et al^[4]^ found in a statistical analysis that 44.2% of patients with taste disorders did not have olfactory disorders and concluded that there is no direct correlation between taste disorders and olfactory disorders.

Although the pathogenesis of taste disorders in patients with COVID-19 is not clear at this time, comprehensive studies have reported that it may be related to angiotensin-converting enzyme 2 (ACE2), a functional receptor for this virus.^[6]^ With the in-depth study of neocoronavirus, ACE2 was found to be highly expressed in the salivary gland, an early target of neocoronavirus SARS-CoV19,^[7]^ and the RNA of neocoronavirus was already present in the saliva before the lung lesions occurred and induced salivary gland dysfunction, affecting the composition and secretion of saliva and leading to taste disorders.^[8]^ Neurological alterations have been suggested as a possible mechanism for how ACE2 contributes to taste disorders in patients with COVID-19.^[9]^ In addition, ACE2 receptors are also expressed in the oral mucosa, and neocoronavirus enters epithelial cells through this channel.^[10]^ The interaction of the virus with Toll-like receptors mediates the inflammatory response and upregulates the levels of inflammatory factors such as *γ* interferon (IFN-*γ*), which causes abnormal turnover of taste bud cells and ultimately leads to the development and progression of taste disorders.^[11]^ Modern medicine has not yet provided targeted treatment for taste disorders caused by COVID-19,^[12]^ which has a serious impact on patients’ mental health and quality of life. In this context, acupuncture therapy has a good application in the clinical treatment of taste disorders caused by different factors. The therapy is diverse, safe and effective, and can effectively relieve the clinical symptoms of taste disorders and shorten the time of taste recovery.

In summary, the causes and mechanisms of the clinical manifestations of taste disorders in patients with COVID-19 are not fully understood.^[13]^ The treatment of such patients is also inconclusive, and Western medical treatment lacks targeted drugs and is mostly based on symptomatic treatment. This, coupled with the unsystematic nature of treatment, has led to a lack of systematic supporting clinical evidence, which to some extent has negatively affected the treatment of this disease. However, acupuncture therapy is only considered as an adjunct and alternative therapy in cases where it has good efficacy. Therefore, in order to objectively understand the efficacy and safety of acupuncture for the treatment of taste disorders due to COVID-19, this study aimed to collect randomized controlled trials of acupuncture for taste disorders and to conduct a complete systematic evaluation and meta-analysis to provide a reliable evidence-based basis for clinical application.

## 2. Methods and analysis

### 2.1. Objectives and registration

The aim of this systematic evaluation is to assess the efficacy and safety of acupuncture in the treatment of post-COVID-19 taste disorders. This systematic evaluation protocol is registered in PROSPERO under the registration ID CRD42022364653. We will follow recommendations out- lined in The Cochrane Handbook of Systematic Review of Interventions and the preferred reporting items for systematic reviews and meta-analysis protocol (PRISMA-P) statement guidelines. We will update our protocols simultaneously to include any changes throughout the study.

### 2.2. Eligibility criteria

The PICOS principles will be referenced to determine the inclusion and exclusion criteria for this systematic review.

#### 2.2..1. *Types of participants*.

COVID-19 posterior taste disorder. There were no restrictions on gender, race, age, country, or stage of disease. A history of trauma, toxic substance exposure, psychiatric factors, and other diseases causing taste disorders were excluded. The diagnosis of novel coronavirus pneumonia and taste disorders included Chinese or international diagnostic criteria.^[14–16]^

#### 2.2..2. *Types of interventions and comparator*.

In addition to the corresponding COVID-19 treatment. For the treatment of taste disorders, the main intervention was acupuncture in the treatment group and comfort therapy (placebo, sham acupuncture or blank control) or other therapies (Western medicine, usual care or non-pharmacological treatment, etc) in the comparison group.

#### 2.2..3. *Types of outcomes*.

In this meta-analysis, recovery of taste function will be assessed by Taste Detection Thresholds,^[17,18]^ and taste function threshold test scores as well as clinical effectiveness will be the primary assessment of this study. Secondary outcomes will assess patient quality of life, satisfaction, and incidence of adverse events.

#### 2.2..4. *Types of studies*.

This study includes only randomized controlled trials (RCTs) that treated post-COVID-19 taste disorders alone or in combination with other interventions. There were no publication language or time restrictions. Non-randomized controlled trials, case reports, clinical experience, and animal studies were excluded.

### 2.3. *Data sources and retrieval strategy*

Randomized controlled trials will be extracted from Cochrane Central Register of Controlled Trials, Embase, PubMed, Web of Science, Chinese National Knowledge Infrastructure, Chinese Biomedical Literature Database, the Chinese Scientific Journal Database and the Wanfang Database. The set period was from the December 2019 to October 2022. All RCTs examining the use of acupuncture in the treatment of taste disorder with COVID-19 will be collected. The main search terms will be “COVID-19,” “Taste Disorder” and “Acupuncture,” and the retrieval formula will be adjusted according to the characteristics of different databases. Taking PubMed as an example, the retrieval strategy is shown in Table 1.

**Table 1:** Search strategy for the PubMed database.

Number	Search items
#1	(“Acupuncture Therapy”[Mesh]) OR ((((((((((Acupuncture Treatment[Title/Abstract]) OR (Acupuncture Treatments[Title/Abstract])) OR (Treatment, Acupuncture[Title/Abstract])) OR (Therapy, Acupuncture[Title/Abstract])) OR (Pharmacoacupuncture Treatment[Title/Abstract])) OR (Treatment, Pharmacoacupuncture[Title/Abstract])) OR (Pharmacoacupuncture Therapy[Title/Abstract])) OR (Therapy, Pharmacoacupuncture[Title/Abstract])) OR (Acupotomy[Title/Abstract])) OR (Acupotomies[Title/Abstract]))
#2	(“post-acute COVID-19 syndrome” [Supplementary Concept]) OR ((((((((((long-COVID[Title/Abstract]) OR (long-haul COVID[Title/Abstract])) OR (post-acute COVID syndrome[Title/Abstract])) OR (persistent COVID-19[Title/Abstract])) OR (post-acute COVID19 syndrome[Title/Abstract])) OR (long hauler COVID[Title/Abstract])) OR (long COVID[Title/Abstract])) OR (post-acute sequelae of SARS-CoV-2 infection[Title/Abstract])) OR (long haul COVID[Title/Abstract])) OR (chronic COVID syndrome[Title/Abstract]))
#3	(“Taste Disorders”[Mesh]) OR (((((((((((((((((((((((Taste Disorder[Title/Abstract]) OR (Taste Disorder, Anterior Tongue[Title/Abstract])) OR (Taste Disorder, Posterior Tongue[Title/Abstract])) OR (Taste Disorder, Primary[Title/Abstract])) OR (Primary Taste Disorder[Title/Abstract])) OR (Primary Taste Disorders[Title/Abstract])) OR (Taste Disorders, Primary[Title/Abstract])) OR (Taste Disorder, Primary, Bitter[Title/Abstract])) OR (Taste, Metallic[Title/Abstract])) OR (Metallic Taste[Title/Abstract])) OR (Metallic Tastes[Title/Abstract])) OR (Tastes, Metallic[Title/Abstract])) OR (Taste Disorder, Primary, Sweet[Title/Abstract])) OR (Taste Disorder, Secondary[Title/Abstract])) OR (Secondary Taste Disorder[Title/Abstract])) OR (Secondary Taste Disorders[Title/Abstract])) OR (Taste Disorders, Secondary[Title/Abstract])) OR (Taste Disorder, Secondary, Bitter[Title/Abstract])) OR (Taste Disorder, Secondary, Salt[Title/Abstract])) OR (Taste Disorder, Secondary, Sweet[Title/Abstract])) OR (Taste Dysfunction[Title/Abstract])) OR (Dysfunction, Taste[Title/Abstract])) OR (Taste Disorder, Primary, Salt[Title/Abstract]))
#4	(randomized controlled trial[Publication Type] OR randomized[Title/Abstract] OR placebo[Title/Abstract])
#5	#1 and #2 and #3 and #4

COVID-19 = coronavirus disease 2019.

### 2.4. Data collection and analysis

#### 2.4..1. Selection of studies.

**Figure 1.**
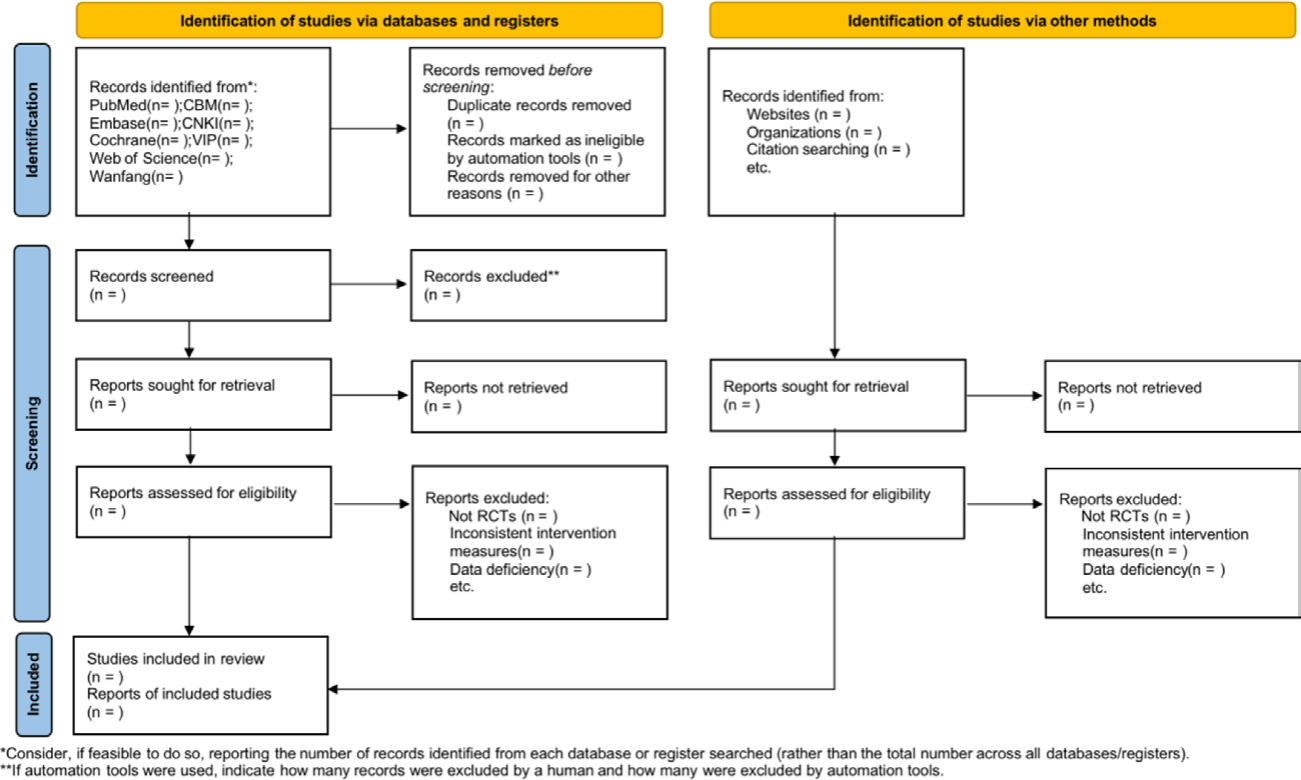
PRISMA flow chart of study selection process.

Two researchers independently searched for relevant research studies and then screened the studies by reviewing titles, abstracts, or full texts as necessary. In addition, if a disagreement arises between the 2 researchers, a 3rd researcher reviews the study. The selection process was summarized using the latest PRISMA flowchart.^[19]^The PRISMA flowchart (Fig. 1) shows the details of the study selection process.

#### 2.4..2. *Data extraction and management*.

Two researchers are responsible for extracting and managing data based on screening results, including study title, journal, year of publication, first author’s name, general information, study design, experimental interventions and timing of interventions, outcomes, and adverse events. Disagreements, if any, will be resolved by consultation with a third researcher.

#### 2.4..3. *Dealing with missing data*.

If the complete literature or relevant data could not be obtained, the corresponding author will be contacted to provide them for us. However, if the missing data were ultimately still not available, the study was excluded from this analysis.

#### 2.4..4. *Assessment of risk of bias*.

Two researchers will assess the quality of included RCTs using the risk of bias assessment tool recommended in the Cochrane systematic review manual 5.1.0. The following aspects will be considered: random sequence generation, allocation concealment, blinding of participants and personnel, blinding of outcome assessments, completeness of outcome data, selective reporting, and other sources of bias. The studies will be rated as “low risk,” “high risk” or “unclear risk.” Disagreements, if any, will be resolved through discussion with other researchers.

#### 2.4..5. *Measure of treatment effect*.

Review Manager (RevMan 5.4, Cochrane Collaboration, Nordic Cochrane Center, Copenhagen, Denmark) software and Stata SE 15.0 (Stata Corp, College Station, TX) will be used to conduct this meta-analysis. Dichotomous outcomes will be presented as RR (risk ratios) with 95% confidence intervals. For continuous variables, the mean difference (MD) or standardized mean difference (SMD) and 95% confidence interval were used as effect scales for combining effect sizes.

#### 2.4..6. *Assessment of heterogeneity*.

Before combining the meta-analyses, the heterogeneity test was conducted using the Homogeneity test (Q test) (the test criterion was *α* = 0.1), and if P>*α*, it indicated that there was no heterogeneity among the studies; otherwise, there was heterogeneity among the studies. The larger the value of *I*^2^, the greater the heterogeneity among the studies. If *I*^2^ ≤ 50%, the heterogeneity is acceptable and the fixed-effect model should be selected for Meta-analysis.

#### 2.4..7. *Assessment of reporting bias*.

If more than 10 studies were included in an outcome indicator, funnel plots and Egger’s test were used to analyze whether there was publication bias. If the funnel plot was symmetrical and the *P* value of Egger’s test was greater than 0.05, there was no significant publication bias among the studies in that group.

#### 2.4..8. *Data synthesis*.

We will use Review Manager (RevMan 5.4) software for data analysis and synthesis. If no significant heterogeneity is observed between studies in each group (*P* ≥ .1 and *I*^2^ ≤ 50%), the data will be processed using a fixed effects model. Meanwhile, if *P* < .1 and *I*^2^ > 50%, a random-effects model will be used.

#### 2.4..9. *Sensitivity and subgroup analysis*.

According to the results of data synthesis, studies with obvious heterogeneity should first apply meta-regression and other methods to find the causes of heterogeneity, and after finding the causes of heterogeneity use subgroup analysis for processing; for studies with obvious heterogeneity but cannot determine the causes of heterogeneity should choose a random-effects model for meta-analysis, and apply sensitivity analysis for reliability analysis of the results to ensure the stability and reliability of the results.

#### 2.4..10. *Quality of evidence evaluation*.

The quality of evidence will be independently assessed by 2 reviewers and graded for recommendation evaluation, development and evaluation. Evidence quality will be rated as “high,” “medium,” “low,” or “very low” according to rating criteria based on 5 parameters (publication bias, inconsistencies, inaccuracies, and research limitations).

#### 2.4..11. *Ethics and dissemination*.

Since this study did not involve patient privacy, ethical approval was not required. Our research results will be published in peer-reviewed journals.

## 3. Discussion

Since the outbreak of the New Coronary Pneumonia epidemic, it has posed a serious threat to the global public health system and has had a profound impact on the productive lives of people worldwide. Taste disorders, a characteristic sign and sequelae symptom of some COVID-19 patients, have seriously affected the quality of life and psychological well-being of patients. However, the mechanism of taste disorder caused by neocoronavirus is still unclear, and the available western medical treatments are limited. As an important component of TCM treatment, acupuncture has certain advantages in the treatment of taste disorders. According to studies, acupuncture can significantly improve olfactory and gustatory functions.^[20]^ However, there is still no corresponding meta-analysis to support the efficacy of acupuncture. Therefore, the purpose of this study was to systematically evaluate the efficacy and safety of acupuncture in the treatment of post-COVID-19 taste disorders and to provide a corresponding evidence-based medical rationale for possible therapeutic options for the treatment of taste disorders.

## Author contributions

**Conceptualization:** Yuqiao Chao, Lili Zhang, Qiaobin Chen.

**Data curation:** Yuqiao Chao, Lili Zhang.

**Funding acquisition:** Shaozong Chen.

**Investigation:** Lili Zhang.

**Methodology:** Biwei Chen.

**Resources:** Yuqiao Chao, Yannan Yu.

**Software:** Yuqiao Chao, Yannan Yu.

**Writing – original draft:** Yuqiao Chao.

**Writing – review & editing:** Qiaobin Chen, Shaozong Chen.
